# Experimental Analysis in Hadoop MapReduce: A Closer Look at Fault Detection and Recovery Techniques

**DOI:** 10.3390/s21113799

**Published:** 2021-05-31

**Authors:** Muntadher Saadoon, Siti Hafizah Ab Hamid, Hazrina Sofian, Hamza Altarturi, Nur Nasuha, Zati Hakim Azizul, Asmiza Abdul Sani, Adeleh Asemi

**Affiliations:** Department of Software Engineering, Faculty of Computer Science and Information Technology, University Malaya, Kuala Lumpur 50603, Malaysia; hazrina@um.edu.my (H.S.); altarturih@gmail.com (H.A.); nasuha@um.edu.my (N.N.); zati@um.edu.my (Z.H.A.); asmiza@um.edu.my (A.A.S.); adeleh@um.edu.my (A.A.)

**Keywords:** Hadoop MapReduce, experiment analysis, fault-tolerance, fault detection, fault recovery

## Abstract

Hadoop MapReduce reactively detects and recovers faults after they occur based on the static heartbeat detection and the re-execution from scratch techniques. However, these techniques lead to excessive response time penalties and inefficient resource consumption during detection and recovery. Existing fault-tolerance solutions intend to mitigate the limitations without considering critical conditions such as fail-slow faults, the impact of faults at various infrastructure levels and the relationship between the detection and recovery stages. This paper analyses the response time under two main conditions: fail-stop and fail-slow, when they manifest with node, service, and the task at runtime. In addition, we focus on the relationship between the time for detecting and recovering faults. The experimental analysis is conducted on a real Hadoop cluster comprising MapReduce, YARN and HDFS frameworks. Our analysis shows that the recovery of a single fault leads to an average of 67.6% response time penalty. Even though the detection and recovery times are well-turned, data locality and resource availability must also be considered to obtain the optimum tolerance time and the lowest penalties.

## 1. Introduction

MapReduce is the most popular data processing model [1], used for Big Data-related applications and services over the cloud. Hadoop is the state-of-the-art industry standard implementation of MapReduce that provides tremendous opportunities to handle data-intensive applications like IoT, web crawling, data mining and web indexing. Hadoop, in addition to with MapReduce, offers flexibility for developers to design their applications in any high-level programming languages. Due to the given flexibility, organisations like Yahoo, Google and Facebook utilise Hadoop MapReduce to successfully manage their data-intensive computations in large-scale computing environments. In addition, Hadoop MapReduce is also used for supporting the implementation of complex algorithms that require high computation power in a distrusted manner such as anomaly analysis, network intrusion detection, and calculating the network centrality [2,3,4]. However, in such environments, faults from a node, service or task are common, and they significantly impact the system performance if the fault-tolerance is not properly handled.

Fault-tolerance is the property of a system that allows consistent operation during faults [5,6]. Hadoop handles fault-tolerance using the master–slave communication through heartbeat messages. If the master node does not receive a heartbeat message from a slave node within a configurable timeout value, the slave node will be labelled as failed. Simultaneously, the successful progress made by the failed node before it fails will be neglected, which incurs a huge waste of processing time and resource usage. Meanwhile, Hadoop must wait for the resource scheduler to assign a free slot to restart the faulty tasks intended to be executed on the failed node for recovery. This problem encourages researchers to optimise the time spent for detecting a fault and recovering from a fault towards achieving minimal performance penalties under faults.

Thousands of hardware and software faults occurred during the first year of Hadoop operation [7]. Code bugs, corrupted data, bad sectors, and out-of-memory faults are also important factors to consider while making the system fault-tolerant. Another study reported that a single tiny hardware fault increases the response time of Hadoop by 39% [8]. Additionally, the non-uniformity or node heterogeneity is also common in today’s environments [9], especially when Hadoop is deployed on a cluster of shared resources. Thus, users are willing to run heavy CPU job requests. The job workload needs virtualised tasks to be spread among the nodes. If other users are running different tasks on these nodes at some point in time, the cluster will experience extreme dynamic behaviour. Consequently, the threats mentioned above must be handled gracefully, otherwise there will be a serious performance violation.

### 1.1. Motivation

To date, experimental studies on the fault-tolerance issues of Hadoop MapReduce have been scarce, as seen in [10,11,12]. Meanwhile, these studies only used the old version of Hadoop that which was not incorporated with the YARN framework, making their experimental results not up to date. Furthermore, the studies have not provided insights on the infrastructure parameters that affect the tolerance conditions under faults and failures. To the best of our knowledge, this is the first research effort that provides an in-depth analysis of the impact of node, service, and task faults, including two main conditions: fail-stop and fail-slow on Hadoop MapReduce response time. This research also investigates the priority of fault penalties and the relationship between fault detection and recovery techniques under various fault circumstances to be considered when designing a fault-tolerance solution. The purpose of this analysis is to address the following research questions:

1. How does the current fault-tolerance in Hadoop MapReduce handle faults when they occur at various infrastructure levels and in different fault conditions?
2. Why do the existing fault detection and recovery techniques in Hadoop MapReduce lead to significant response time penalties?

### 1.2. Our Contributions

The main contributions of the paper are the following:

- We conducted a series of experiments on a real-world Hadoop YARN cluster to examine the impact of fail-stop and fail-slow when they occur at the node, service, and task;
- We simulated actual production faults: fail-stop and fail-slow to be injected at runtime and monitor their implications for the response time;
- We highlight the limitations of the current fault-tolerance method, including its fault detection and recovery techniques.

### 1.3. Paper Organisation

Section 2 reviews the related literature, including experimental studies and proposed fault detection and recovery techniques in Hadoop MapReduce. Section 3 provides detailed information on Hadoop MapReduce and fault-tolerance. Section 4 shows the experiment setup and the evaluation parameters, followed by the analysis and discussion of the results in Section 5. Section 6 concludes the paper.

## 2. Related Works

### 2.1. Experimenting with Hadoop Fault-Tolerance

Faghri et al. [11] proposed a model called failure scenario as a service (FSaaS) to be utilised across the cloud to examine the fault-tolerance of cloud applications. The study focused on Hadoop frameworks to test real-world faults implications on MapReduce applications. Kadirvel et al. [10] and Dinu and Eugene Ng [12] conducted experimental studies to examine the performance penalties under faults using an experimental testbed of Hadoop frameworks. These studies mainly focused on node and task faults, but they used the early implementation of Hadoop that was not integrated within the YARN framework, making their experimental results outdated. In the early version of Hadoop, the responsibility of data processing was carried by two components, namely JobTracker and TaskTracker. Subsequently, YARN was designed to allow flexibility and scalability by separating the resource management functions from the programming model [13]. Thus, YARN becomes the essential resource management framework for Hadoop implementation. Furthermore, a recent study on the implications of faults on MapReduce applications by simulating node and task faults proposed by Rahman et al. [14]. Although [14] is quite similar to our study, it has not provided perception when modifying the fault-tolerance parameters offered by Hadoop frameworks and has not focused on the correlations between the fault detection and recovery stages. Unlike the related works, our study uses the latest implementation of Hadoop to cope with its new architectural changes and provides detailed analysis on the common types of faults with their implications. We also examined the tolerance time when modifying the default fault-tolerance parameters to confirm the problem.

### 2.2. Improving Fault Detection

LATE [15] and SAMR [16] were first proposed and adopted in the current speculative execution strategy of Hadoop. These strategies work by comparing the estimated time to completion between tasks; then, the detected struggler tasks will be duplicated on another node. The estimated completion time in LATE was static; thus, SAMR provides a dynamic calculation of the progress rate for each task to achieve more accurate results. In another study, Memishi et al. [9] also proposed an approach that estimates the completion time of the workload and calculates the progress rate of each task to adjust the timeout value dynamically. Other studies by [17,18,19,20] provided predictive models based on machine learning and AI algorithms to estimate and set an optimal heartbeat timeout on the fly or to predict the failures before they occur. These approaches reduce the task fault occurrences and improve their overall performance with low latency in fault detection. Furthermore, works by Yildiz et al. [21] and Kadirvel et al. [22] aimed to decrease resource usage during failures by adopting a lightweight pre-emption technique and dynamic resource scaling to reduce the cost of the additional resources when removing failures.

### 2.3. Improving Fault Recovery

The standard solution to address the problem of re-computing the entire data block from the beginning during fault recovery is checkpointing. Checkpointing works by transferring the continuous processing output to external storage to be restored in the event of failures. In MapReduce, the output of MT tasks is stored locally on the same node, which will be inaccessible when the node encounters a fail-stop. Therefore, research works by [23,24,25] proposed checkpointing algorithms to efficiently transfer the output to external storage to avoid faulty task re-execution from scratch. Although the proposed checkpointing algorithms improved the recovery process when the failed tasks are rescheduled on a different node, the re-computation still occurs because the stored checkpoints are not accessible by active nodes in the cluster. Zhu et al. [24] designed a novel fault-tolerance strategy that uses a combination of distributed checkpointing and a proactive push mechanism for low latency recovery. When a failure happens, the recovered task continues computing based on the last checkpoint without the necessity to re-compute the entire data block. Liu et al. [25] made further improvements by reducing the task recovery delay and improving the processing efficiency. The proposed approach splits the intermediate data into small piece groups instead of merging them into one single file. The recovery task attempt can start with a specific amount of progress according to the valid checkpoint generated along with spills.

## 3. Faults in Hadoop MapReduce

**Figure 1:**
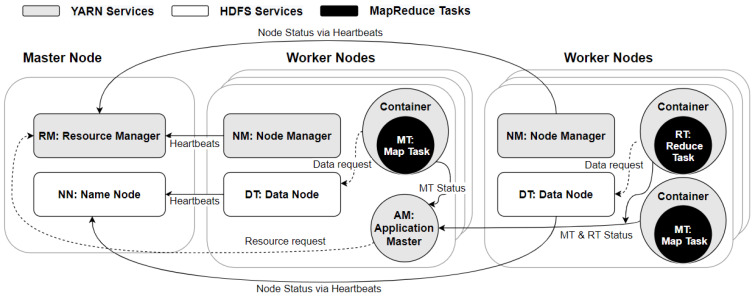
Interactions between HDFS, YARN and MapReduce frameworks with their components.

In this work, we used Apache Hadoop 2.9.2 (https://hadoop.apache.org/docs/r2.9.2/ (accessed on 3 May 2021)), which is the stable implementation of MapReduce that has adopted as the latest generation of MapReduce with YARN framework [13]. Hadoop MapReduce is designed based on the centralised architecture in which one node stands as the master node and other nodes are workers. The basic components of Hadoop MapReduce implementation are HDFS, YARN and MapReduce, as presented in Figure 1. First, HDFS [26] splits the original dataset into data blocks and distributes them amongst its distributed file system that is managed by DN services. The size, replication and distribution of the blocks are handled by the master node of HDFS called NN. Then, YARN is also a centralised framework responsible for workload scheduling and resource management. YARN operates RM and Scheduler on the master node and NM, AM, and Containers on the worker nodes. RM along with the Scheduler maintains the resource scheduling and monitoring of worker nodes. RM launches NM on each worker node to offer a collection of physical compute resources such as (memory and CPU) in the form of containers for handling MapReduce applications. NM is also liable for sending heartbeat messages to RM to report the worker nodes’ liveness.

Furthermore, AM has the responsibility to negotiate appropriate resources for containers and monitor MapReduce tasks isolated in containers. On the other hand, MapReduce is a parallel data processing model that consists of the map phase, shuffle phase and reduce phase. map and reduce are the central progressing aspects of MapReduce that make the key/value pairs data structure. All the MTs are distributed and executed in parallel in YARN containers. During these processes, the intermediate output of every complete task will be generated in the local storage of each node. Then, the output files are shuffled and stored to be finally received by the corresponding RTs. The reduce phase stores the desired output on HDFS.

In parallel systems, three categories of fault models are primarily considered, which are fail-arbitrary, fail-stop and fail-slow [27]:

- Fail-arbitrary is also called byzantine, which impacts the system’s behaviour by setting incorrect data values, returning a value of incorrect type, interrupting, or taking incorrect actions;
- Fail-stop causes unresponsive behaviour that is continuous for a fixed period [28];
- Fail-slow is also known struggler, which makes the system accessible, but with poor performance [29].

Hadoop MapReduce tolerates fail-stop and the fail-slow while fail-arbitrary is beyond the original design of MapReduce [6]. Fail-stop and fail-slow typically occur in one or more components at different infrastructure levels. Therefore, fault-tolerance techniques can be applied to tolerant faults and are fundamentally conducted based on two main stages: fault detection and fault recovery [30].

### 3.1. Fault Detection

**Figure 2:**
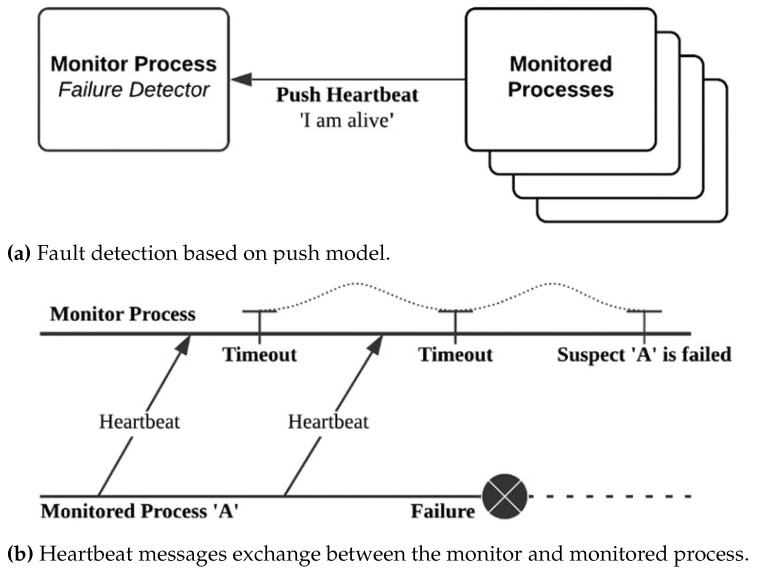
Fault detection in Hadoop MapReduce.

Fault detection is the first building block of a fault-tolerant framework that desires to detect faults as soon as they occur [31]. Hadoop MapReduce uses heartbeat monitoring based on the push model as a default approach. As illustrated in Figure 2a, all the monitored processes periodically send heartbeat messages at a specific timeout frequency to the monitor process. The absence of heartbeats from a given process beyond the specified timeout value indicates that the process is failed, as shown in Figure 2b. The heartbeat timeouts in Hadoop MapReduce are defined as configurable parameters [32], discussed in Section 4.

Although push-based monitoring is a scalable approach as it occupies less bandwidth due to the small size of the heartbeat messages [31], it has major limitations. First, it only indicates possible fail-stop, whereas a process with fail-slow may still send heartbeats. Second, achieving an optimum timeout is challenging because the longer the timeout is, the longer the MTTD and the MTTR. In contrast, the shortest timeout is the shortest MTTD, however, it may decrease the performance and increase resource consumption (e.g., bandwidth and CPU usage) because of the elevated messages exchange between the active processes when the timeout value is small. Hence, configuring the timeout impacts the overall response time and availability.

### 3.2. Fault Recovery

Upon fault detection, recovery is the second major stage that aims to return a faulty component to its normal state without service interruptions. From the data storage perspective, HDFS replicates the original data blocks into multiple copies to be stored on various nodes. A replica number determines the replication in HDFS. For instance, storing 1TB of data requires 3TB of actual storage space when the number factor is set to 3. Although data replication is lacking in terms of storage efficiency [33], it maintains high fault-tolerance and data availability. Furthermore, a simple fault recovery technique is implemented for handling task faults, i.e., the re-execution or restart upon failures. This approach effectively recovers fail-stop tasks by rescheduling them to re-execute their attended functions from the very beginning. However, the re-execution technique doubles the workload execution time, especially when the task fails to process the final allocated data record. Then, an additional fault recovery approach is also employed, which is checkpointing [34]. This approach aims to efficiently recover from faults with minimal performance overhead. Hadoop separates MT and RT into two categories: complete and incomplete. In the event of failure, the complete tasks do not have to start over because their outputs are transmitted to the next task that could be hosted on another healthy node. However, when the incomplete tasks are in the latest progress rate and a fault occurs, Hadoop starts them over just like the incomplete tasks regardless of their progress, which will lead to excessive performance overhead. Furthermore, Hadoop recognises fail-slow as slow/struggling tasks by comparing their progress rate with other active tasks. When Hadoop realises them, speculative attempts will be made to process the same input data block of each slow task, hoping that these attempts are complete sooner than the slow ones.

## 4. Experiment and Evaluation

The evaluation experiments aimed to analyse the current fault detection and recovery techniques of Hadoop MapReduce, focusing on the performance violation in terms of response time when tolerating faults and failures. Based on the analysis, further study was conducted to examine the detailed impact of the infrastructure parameters, fault types and fault-tolerance conditions.

### 4.1. Experiment Setup

**Figure 3:**
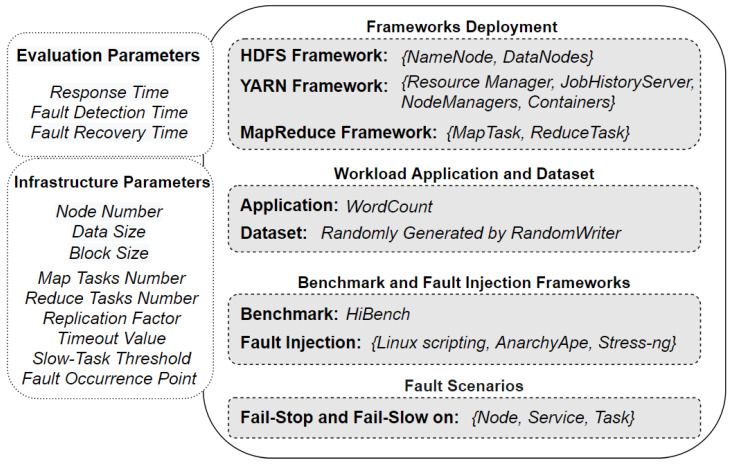
The main parts of the experimental testbed.

Our experimental testbed consisted of 9 servers that ran 1 master node and 8 slave nodes. The master node had an 8-core CPU, 80 GB of hard disk space and 16 GB of RAM. Other nodes consisted of a 4-core CPU, 40 GB of hard disk space, and 8 GB of RAM. These nodes were hosted on a Cavium server with ThunderX 88XX 48CPUs, 128 GB RAM and 3 TB of hard disk space in total. All the nodes operated on CentOS7 and Hadoop with HDFS, YARN and MapReduce frameworks installed. Figure 3 shows our experiment setup divided into four parts as discussed in the following subsections.

#### 4.1.1. Workload Application and Dataset

Since MapReduce is a general programming paradigm, a very diverse set of applications can be constructed using the basic map, shuffle/copy, and reduce phases. To ensure our analysis was applicable in the experiment environment, we used WordCount benchmark application as it includes all the basic phases of typical MapReduce programming paradigm [35]. WordCount is a count value for every distinct word in the input dataset where the count function is demonstrated by MTs [10]. WordCount is not only used as a benchmark application, as observed in [10,11,12,14], but it is also being used in production data processing environments. For instance, WordCount represents 70% of production jobs in the Facebook cluster [36]. Furthermore, most workloads in production clusters >98% had small to medium execution times (seconds to a few tens of minutes) as noticed in Yahoo! and Facebook studies [35]. Therefore, we used 1 GB to 8 GB randomly generated datasets to represent a small to medium-workload size and have execution times of a few minutes.

#### 4.1.2. Benchmark and Fault Injection Frameworks

Our experiments were automated based on frameworks and techniques for performance monitoring and fault injection. First, we used HiBench (https://github.com/Intel-bigdata/HiBench (accessed on 10 April 2021)) as the standard benchmark framework for implementing MapReduce jobs and monitoring the performance of each job. Intel developed HiBench as an open source project, and it has been used for evaluating Big Data related systems, including Spark and Hadoop MapReduce. Second, we used manual Linux scripts and popular cloud-based fault-injection frameworks for fault injection, namely AnarchyApe (https://github.com/david78k/anarchyape (accessed on 10 April 2021)) and Stress-ng (https://github.com/ColinIanKing/stress-ng (accessed on 10 April 2021)). These frameworks provide various options for simulating faults in the Hadoop environment, as outlined in Table 1.

In the experiments that involve various fault occurrence points, we group the fault points according to the primary MapReduce phases; before 20% of the map phase progress is ’Initial’, after 20–50% as ‘Early’, before 70% as ‘Middle’ and those after 70% as ‘Late’. In the case of the late point, the fault impacts the active reduce tasks only, while the fault at the middle point impacts both the map and reduce tasks. Fail-stop and fail-slow for node, service and task are injected for a fixed period until Hadoop realises them and applies treatment actions for recovery. Moreover, since the replication factor is set to two where one data block is at least available on two active nodes, we injected one fault of different types (node fail-stop, service fail-stop, task fail-stop and fail-slow) at each experimental run.

Furthermore, the monitoring of fault detection and recovery stages was performed via manual invocation of Hadoop log files after each job completed. The manual exploration process was necessary for the flexibility of data collection of the history files generated by the nodes with their active services and tasks. Parsing the generated data indicates the timestamp when Hadoop detects the fault and the timestamp for initiating the recovery action. Since the faults are injected at known points, the fault detection and recovery times can be calculated accordingly.

#### 4.1.3. Fault Scenarios

**Figure 4:**
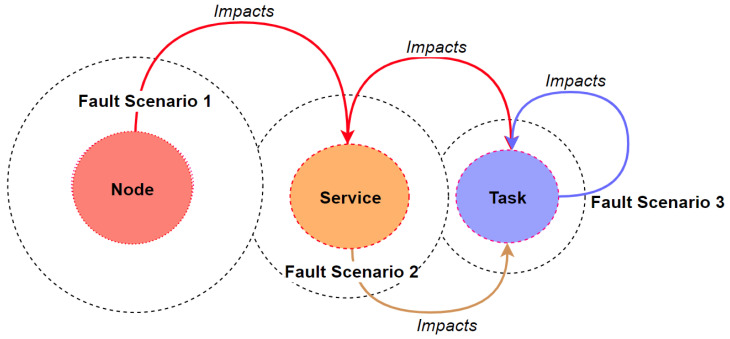
Fault scenarios in Hadoop MapReduce.

We classify the faults according to their occurrence into three scenarios: node, service, and task, as demonstrated in Figure 4, when the entire node fails due to a certain memory, CPU, or network error, all the running services and tasks hosted by the failed node are impacted, and the tolerance complexity increases. Likewise, when a service fails, all its associated tasks fail as well and are subject to re-execution. Moreover, if a single task fails, Hadoop intends to re-execute it on the same or another active node. With those three scenarios, we cover all the possible cloud-based fault and failure implications due to either fail-stop or fail-slow.

### 4.2. Evaluation Parameters

To measure the performance of Hadoop MapReduce under faults and failures, we use three main evaluation parameters as explained in the following subsections. Moreover, we also use nine configurable parameters provided by Hadoop frameworks that impact the job response time and the tolerance time in the event of failure, as briefly described in Table 2. Finally, we focus on the timeout value and the slow-task threshold in Section 5.2 to show the influence of fault recovery when modifying the default fault detection parameters. The default and tuned values of the fault detection parameters are outlined in Table 3.

#### 4.2.1. Response Time

Response time is the time taken by the MapReduce job from submission $J_{s}$ to completion $J_{c}$ and it can be calculated according to Equation (1):

(1) $$Response\;time=J_{c}\;-\;J_{s}$$

#### 4.2.2. Fault Detection Time

Fault detection time is the time taken by the Hadoop MapReduce to detect a fault from the fault occurrence $F_{o}$ timestamp to the fault detection $F_{d}$ timestamp, and it can be calculated according to Equation (2):

(2) $$Fault\;detection\;time\;=F_{d}\;-\;F_{o}$$

#### 4.2.3. Fault Recovery Time

Fault recovery time is the time taken by the Hadoop MapReduce to recover from a fault from the fault detection $F_{d}$ timestamp to the fault recovery $F_{r}$ timestamp, and it can be calculated according to Equation (3):

(3) $$Fault\;recovery\;time\;=F_{r}\;-\;F_{d}$$

## 5. Results and Discussions

### 5.1. Response Time

The first series of experiments investigated the impact of faults and failures on the response time when modifying the infrastructure parameters; (a) increasing the dataset size, (b) setting the different distribution of data blocks among the nodes, and (c) injecting faults at various occurrence points during the execution time. We used the default fault-tolerance values provided by the framework, which are 600 s expiry interval timeout and 1.0 slow task threshold. Fail-stop are simulated by permanently killing an active node, service/daemon (e.g., NM, DN or AM), MT or RT. We also used AnarchyApe, and Stress-ng to inject fail-slow such as CPU hog, Memory hog and network drop packets. Throughout our experiments, Hadoop recovered from all the injected faults and failures at various response times.

**Figure 5:**
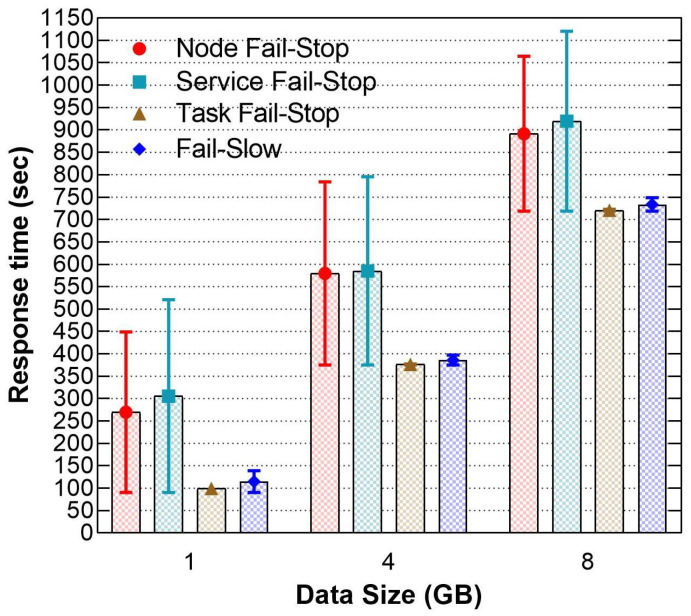
The response times differences when increasing dataset size.

Figure 5 shows that the service fail-stop has led to the highest response time penalties all over the experiments. The absence of a service does not impact the parallelism nor the MT progress among the nodes. However, when the scheduler launched the RT, the entire job is suspended because there were no data pushed from the impacted node, even though the hosted MTs were completed successfully. Furthermore, the scheduler has not triggered a speculative task for recovery because the progress of all the running MTs was identical at that moment. In addition, the RM waited until the next timeout cycle expired to confirm the service has failed to restart all the impacted tasks associated with the failed service on another healthy node from scratch. In brief, the response time of the impacted MTs were doubled, and the total response time extended per each healthy task workload.

Then, the entire node was forcibly terminated from the cluster, including its active services and tasks (NM, DN, MT, or RT) at runtime to evaluate node fail-stop. The loss of a node leads to network disconnection where the input data provided by HDFS became unreachable. Thus, the progress rate of the running tasks stopped at the exact moment when the failure occurred. Thereafter, the scheduler detected the impacted tasks and restarted them on another healthy node. Therefore, the response time penalties for node fail-stop are slightly lower than the service fail-stop because the RM depends on the successive heartbeats (by default 600 s) to be received from the failed node to finish the job, and the scheduler had already re-executed the impacted tasks before the heartbeat timeout expired. Furthermore, task fail-stop and fail-slow incurred the lowest penalties in all the cases because Hadoop scheduler speculates the impacted tasks on another node once it detected they are terminated, or their progress rates are lower than the other active tasks without relying on the node expiry timeout.

**Figure 6:**
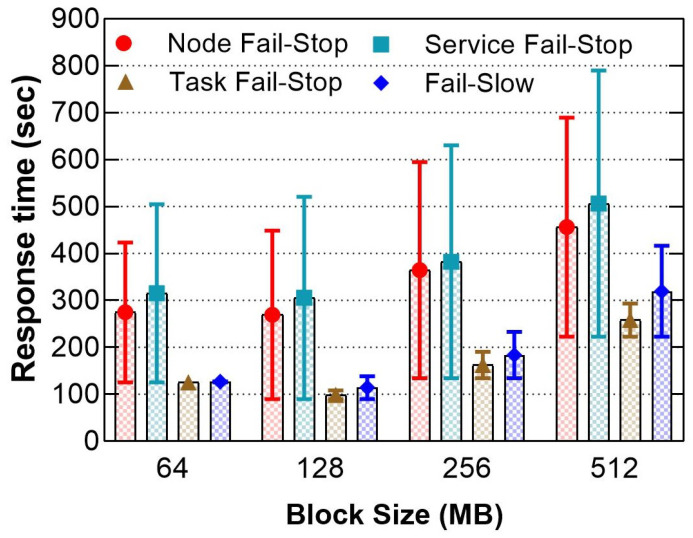
The response times’ difference when setting a different distribution of data.

Figure 6 compares the response times when using various distributions of data blocks among the cluster. By default, HDFS splits the dataset into multiple blocks, and each block has 128 MB of data to be distributed on the active DNs. For instance, 1 GB of data is divided into 8 blocks of 128 MBand multiplied by the replication factor. We used Equations (4) and (5) to obtain accurate results and ensure an equivalent load of tasks among the active nodes in the cluster, where each task corresponds to a data block:

(4) $$Number\;of\;blocks=\frac{data\;size}{block\;size}\;\times replication\;factor$$

where the task parallelism will be set as follows:

(5) $$Task\;parallelism\;=\frac{Number\;of\;blocks}{replication\;factor}$$

The result reveals that the service and node fail-stop still have a higher response time penalties than task fail-stop and fail-slow. The result also shows that task-fail-stop and fail-slow incurred greater penalties when the block size increases. The penalty confirms that when the MT processing time increases due to a large block of data, Hadoop spends longer time recovering from the failure due to the restart. As a result, one task failure extended the overall job response time by 29.47%, 31.66% for the worst-case of 256 MB, 512 MB block sizes, respectively.

**Figure 7:**
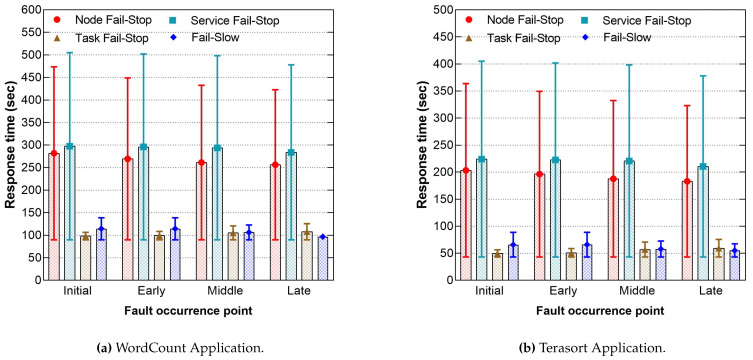
The response times’ differences when injecting faults at various occurrence points for WordCount and Terasort Application.

Figure 7 compares the injected faults at various occurrence points at runtime. Furthermore, it shows the response time differences between WordCount and Terasort applications when faults accumulated initially, and at the early, middle, and late points of the job lifetime, as recorded in Figure 7a,b. The tolerance time for the node and service fail-stop slightly decreased when they occur late because both depend on the master node to wait for the next heartbeat cycle to detect them and start the recovery actions. Since the fault-free job for both WordCount and Terasort had only one timeout cycle, faults at the late point of the timeout period required a shorter time to complete the cycle. On the other hand, task fail-stop penalties increased by 18.17%, 34.27% for the late point compared to the initial because the entire tasks waited for the re-execution of the faulty task from the beginning. However, the fail-slow response time penalties decreased at the late points by 25.4%, 23.94%, because the impacted node only suffered slow performance for a few seconds before it was completed without triggering the speculative task. The results also show no significant difference in terms of fault-tolerance when using various MapReduce applications because the current fault-tolerance method does not consider the programming logic of the applications to detect and recover the faults and failures.

### 5.2. Fault Detection and Recovery Times

The second series of experiments was conducted to investigate the fault detection and recovery time when manipulating the default fault-tolerance parameters provided by the frameworks. The parameters involved the expiry timeout values of the node, service and task.

**Figure 8:**
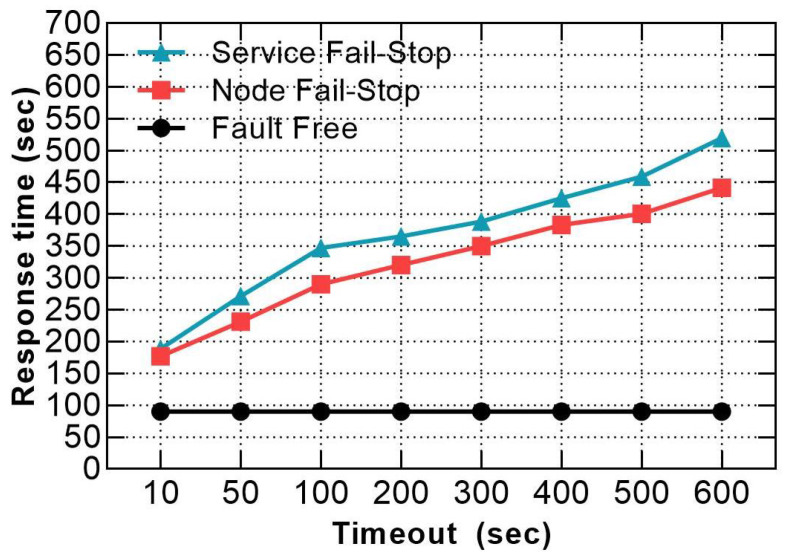
Comparison between node fail-stop and service fail-stop response times when using various timeout values.

**Figure 9:**
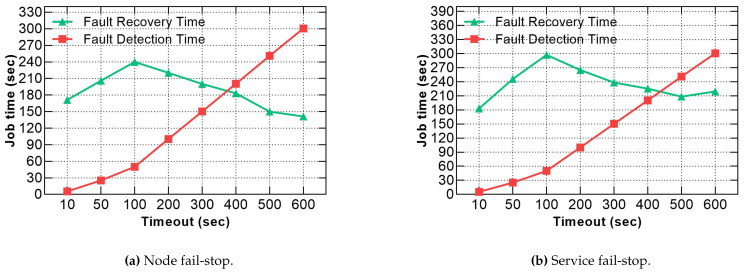
Comparison between fault detection and recovery times for node and service fail-stop using various timeout values.

Figure 8 shows that the response times for both node and service fail-stop were remarkably decreased by 60% and 63.71%, respectively, in the case of 10 s timeout compared to the default value of 600 s. Although the optimal response time results incurred at the smallest timeout value, Hadoop recovery procedure took 171 s, 183 s to recover from node and service fail-stop, respectively, which is still very slow, as reported in Figure 9a,b. Moreover, if Hadoop detects the fault in a very short time, it needs a longer time for recovery, especially when the cluster runs on full resource capacity because the scheduler waits until allocating available slots to re-execute all the impacted tasks of the faulty node or service. Furthermore, reducing the timeout value for obtaining a rapid detection time is not an ideal option to achieve minimal response time penalties because a short timeout incurs a higher resource consumption [10], due to the elevated messages’ exchange between the active processes in the cluster in a short frequency.

**Figure 10:**
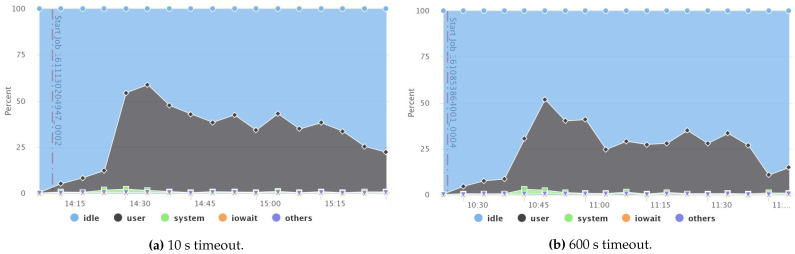
Comparison between CPU usage when using small and default timeouts.

**Figure 11:**
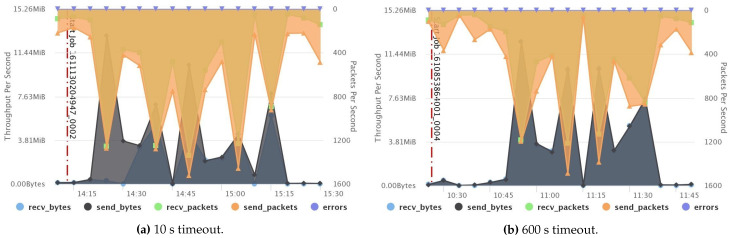
Comparison between network usage when using small and default timeouts.

**Figure 12:**
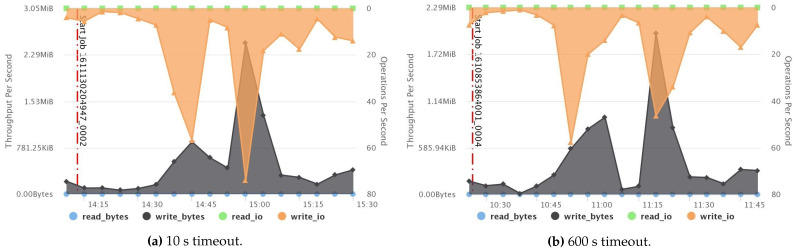
Comparison between disk usage when using small and default timeouts.

**Figure 13:**
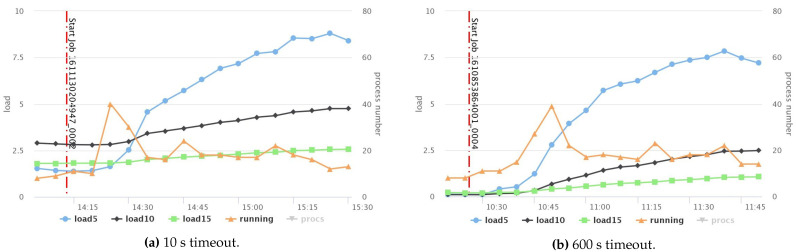
Comparison between system loads when using small and default timeouts.

Figure 10a,b show a comparison of the CPU summary usage between two fault-free jobs that have two different timeout values: 10 s (small) and 600 s (default), respectively. The result reveals that the small timeout value incurred higher CPU usage than the default one. Likewise, network throughput, disk throughput, and the overall system load also acquired higher usage, as recorded in Figure 11, Figure 12 and Figure 13, respectively. However, short timeouts could be an acceptable approach for specific use cases when the application must comply with response time constraints and sacrifice resource consumption.

**Figure 14:**
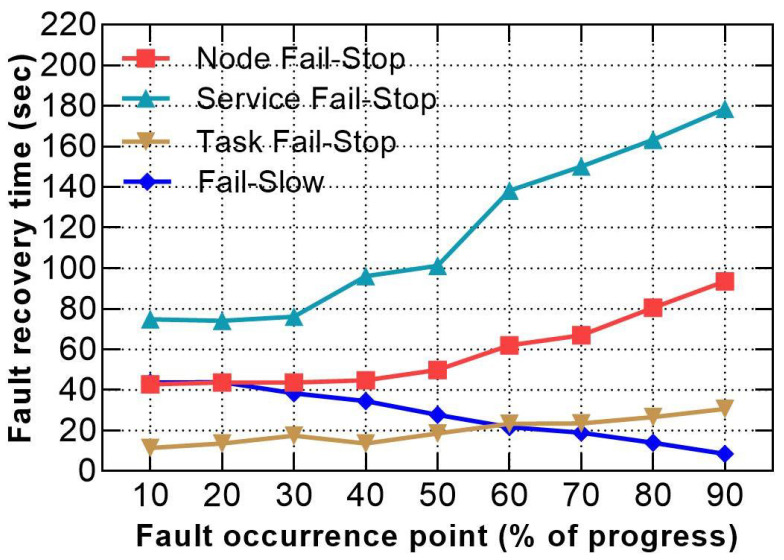
Recovery times when using small timeout and injecting faults at various points.

Finally, we intend to observe the minimal recovery time when setting aggressive slow-task threshold https://hadoop.apache.org/docs/r2.9.2/hadoop-mapreduce-client/hadoop-mapreduce-client-core/mapred-default.xml (accessed on 10 April 2021) of 0.1 and timeout values https://hadoop.apache.org/docs/r2.9.2/hadoop-yarn/hadoop-yarn-common/yarn-default.xml (accessed on 10 April 2021) of 10 s and injecting the faults at various occurrence points of the job lifetime regardless of the resource consumption. Figure 14 shows that even though the detection time is optimised to approximately 5 s and the response times are reduced compared to previous scenarios, the average recovery time for Hadoop is 55.84 s for a workload of 90 s. A comparison between the best, median and extreme values in terms of fault recovery time, fault point and response time penalty is recorded in Table 4.

### 5.3. Discussion

According to the obtained results, a single faulty task extends the overall job response time by 30%, and even though the framework was well tuned for fault-tolerance, the faulty task incurs an 18.24% response time penalty after recovery. We also confirmed experimentally that this penalty was due to the slow fault detection and the waste of resources by fault recovery in the typical Hadoop fault-tolerance. We summarise our findings based on the experiment results as follows:

- Service fail-stop has the highest response time penalties compared to the other faults;
- When the size of a task’s workload increases due to a large data block, the recovery time also increases because of the re-computation of the entire block;
- Fault occurrence at the late point of the job lifetime incurs higher penalties for node and service fail-stop and lower penalties for task fail-stop and fail-slow;
- The current fault-tolerance method does not consider the programming logic of the application to detect and recover faults and failures;
- The response time decreases when setting small timeout values but with higher resource consumption;
- The recovery of a single fault leads to an average of 67.6% response time penalty.

We answer the research questions 1 and 2 stated in Section 1.1 in the following:

1. The current fault-tolerance method of Hadoop handles fail-stop and fail-slow from node and service based on the static heartbeat messages and re-execution techniques. If the entire node fails, all its active services and tasks fail as well regardless of their progress, and they will be subject to re-execution. The identification of fail-stop only happens when the node is entirely inactive within a static timeout interval. When a service fails, Hadoop is not able to realise the failure because the master node still receives heartbeat messages from the same node that runs the failed service, which leads to a substantial waiting time. Fail-slow has a direct impact on the task level only, and Hadoop detects task fail-slow and fail-stop by comparing the slow task progress with other healthy tasks. Slow tasks are also subject to restart from scratch on another node and based on the scheduler decision in terms of data locality and resource availability.
2. The significant response time penalties happen because of the static waiting time spent by Hadoop to detect a failure. This waiting time is critical because if one node fails out of thousands, the entire job response time is extended per one node fault detection and recovery times. Even though the detection time is optimised based on short monitoring intervals, the recovery time can still be long because it depends on the cluster behaviour in the recovery stage in terms of resource capacity and the locations of data blocks.

In summary, the limitations of fault-tolerance capability are due to the centralised manner in which Hadoop applies fault detection and recovery. In this way, the timeout is hard to set dynamically to mitigate the fault detection problem because it repeatedly requires examining the unpredictable behaviour of all the active nodes in the cluster. On the other hand, we argue that although the generated data by MTs and RTs can be checkpointed and distributed for faster recovery, this method still leads to network and I/O delays using the current centralised architecture because of checkpoints transfers from one node to another [25].

## 6. Conclusions and Future Works

Hadoop MapReduce has been widely used by business and academia sectors due to the scalability and the built-in fault-tolerance capability. However, Hadoop experiences numerous types of faults which must be handled carefully; otherwise, there will be a severe response time violation. In this study, we provide an in-depth analysis of the implications of node, service, and task faults, including the two main conditions: fail-stop and fail-slow on Hadoop MapReduce response time. We simulated actual faults that happen in any distributed environments based on popular fault injection frameworks. We also conducted a series of experiments on a real-world Hadoop cluster to examine the fault-tolerance problem and highlight the limitations, including the fault detection and recovery techniques. The validity of our experimental results is limited to small to medium workload execution times where the size of datasets ranges between 1 GB and 8 GB. Although most workloads in production clusters run MapReduce applications for seconds to a few tens of minutes execution times, more computation resources are necessary to handle the scale and to extend the validity of the experiments.

In future works, we intend to design and implement a new fault-tolerance method based on an explicit node-to-node relationship to address the identified limitations. With this relationship, the timeout values can be set independently for every two pairs of nodes for faster fault detection, rather than being solely controlled by the master node. This way, the scalability and resource efficiency can be further improved because the timespan by which a node checks the status of its pair is not affected by the total number of nodes in the cluster. On the other hand, since the checkpointing approach has been proven in improving the efficiency of fault recovery in Hadoop, we intend to apply an in-memory distributed database to the checkpoint and transfer the output of the active nodes after each heartbeat message being sent to guarantee a consistent progress rate of two running instances and to prevent any wasting the processing progress in the case of failures. The network and I/O delays would be decreased as the pairs will be pre-defined before executing the jobs where the data locality and resource availability can be considered.

## Figures and Tables

**Table 1:** Samples of fault options used for fault injection.

Framework/Techniques	Fault Type	Command
AnarchyApe	Node fail-stop	java -jar ape.jar -L -F
AnarchyApe	Service fail-stop	java -jar ape.jar -L -k <serviceName>
Manual script	Task fail-stop	Sudo kill -9 <processID>
Stress-ng	Fail-slow	stress-ng –cpu 8 –io 8 –vm 1–vm-bytes 16G –timeout 150 s

**Table 2:** Brief description of the infrastructure parameters used in the experiments.

Infrastructure Parameters	Description
Node number	The number of the active nodes in the cluster.
Data size	The size of input data to be processed by the active nodes.
Block size	The size of each chunk of data after distribution across the nodes.
Map task number	The total number of map tasks to be executed on the active nodes.
Reduce task number	The total number of reduce tasks to write the output on HDFS.
Replication number	The number of replicas per each data block.
Timeout value	The time difference between each heartbeat message sends to check the instance liveness.
Slow-task threshold	The standard deviations number for a task average progress.
Fault occurrence point	The actual timestamp of injecting a fault during the job lifetime.

**Table 3:** Configurable fault detection parameters provided by Hadoop MapReduce.

Fault Detection Parameter	Default Value	Tuned Value	Description
yarn.nm.liveness-monitor.expiry-interval-ms	600 s	10 s	Time in seconds to wait until considering the NM dead.
yarn.nodemanager.health-checker.interval-ms	600 s	10 s	Frequency of running node health script.
mapreduce.task.timeout	600 s	10 s	Time in seconds before a task will be terminated if it neither reads an input, writes an output, nor updates its status string.
mapreduce.job.speculative.slowtaskthreshold	1.0	0.1	Standard deviations number by which a task average progress must be lower than the average of all running tasks.

**Table 4:** Comparison between the best, median and extreme values in terms of fault recovery time, fault point and response time penalty when setting optimal fault-tolerance parameters.

Group	Fault Type	Fault Point (%)	Recovery Time (sec)	Response Time Penalty (%)
Best	Node fail-stop	10	42.73	53.03
	Service fail-stop	10	74.911	88.79
	Task fail-stop	10	11.414	18.24
	Fail-Slow	90	8.46	14.96
Median	Node fail-stop	50	49.927	61.03
	Service fail-stop	50	101.241	118.05
	Task fail-stop	50	18.632	26.26
	Fail-slow	50	27.678	36.31
Extreme	Node fail-stop	90	93.503	109.45
	Service fail-stop	90	178.555	203.95
	Task fail-stop	90	30.749	39.72
	Fail-slow	20	43.798	54.22

## Abbreviations

The following abbreviations are used in this manuscript:

HDFS	Hadoop Distributed File System
YARN	Yet Another Resource Negotiator
MT	Map Task
RT	Reduce Task
RM	Resource Manager
NN	Name Node
NM	Node Manager
DN	Data Node
AM	Application Master
MTTD	Mean Time to Detect
MTTR	Mean Time to Repair
